# Addressing the theory-practice gap in assessment

**DOI:** 10.1007/s40037-016-0323-z

**Published:** 2017-01-03

**Authors:** Sarah R. Wright, Matt Homer

**Affiliations:** 1 Michael Garron Hospital and University of Toronto, Toronto, Ontario Canada; 2 University of Leeds, Leeds, UK

This issue of Perspectives on Medical Education contains a thought-provoking article by Young, Cummings & St-Onge, looking specifically at the stability of difficulty and discrimination indices when calculated for small samples of students. They concluded that difficulty and discrimination scores calculated based upon small samples are not stable across cohorts and therefore should be interpreted with caution [1]. Given our mutual experiences in roles that seek to implement theoretically-based and research-informed assessment practices, this article prompted us to think about assessment processes *as a whole*. We believe holistic review of assessment processes and practices is foundational to generating meaningful interpretations of individual metrics, such as difficulty and discrimination indices. In this commentary, we argue that assessment research would benefit from improved integration of theory and practice.

The intersection of theory and practice can be tricky ground to navigate. Theoretically, an assessment strategy might be ideal, but impossible or impractical to implement. For example, despite their imperfections, there is theoretical support for the use of (modified) Angoff [2] and Ebel [3] standard setting methods. However, the implementation of these test-based standard setting methods requires someone to advocate for, hold and lead meetings, collect the results of the meeting and have the expertise to use the results to set a cut score. In addition, a team of clinical teachers must attend the meeting, be familiar with the assessment and course content and have a level of understanding of the concept of the minimally competent student [4]. Assessment items must be readily available to discuss at the meeting, ideally stored in a question bank. Remediation strategies must be in place for students who have not met the standard. While these methods have a sound theoretical base, medical schools may not use them due to issues of feasibility.

Conversely, systems may be in place that are very practical, but have no theoretical underpinning. For example, it is not uncommon for medical schools to use a long-standing pass mark for all examinations; Young & colleagues are certainly not alone in using 60% as the standard pass mark, regardless of the difficulty of the examination. While this may be an institutional standard, philosophically, this strategy is difficult to support [2, 5]. Assessment research becomes quite difficult to conduct and interpret when little is known about how assessments were designed, how quality is ensured on an ongoing basis, and how pass marks are generated.

Why does this assessment theory-practice gap occur? While researchers might have theoretical assessment knowledge, they are not likely to express a desire to be more involved in the operational side of assessment, which may be deemed ‘service work’ in positions that primarily reward basic, rather than applied, research activity [6]. Likewise, clinical teachers who are involved in the creation and delivery of assessments do not always have the required assessment expertise or time to advocate for best practices. Furthermore, if they are given one specific area of the curriculum to teach and assess based on their specialty area of expertise, they are not best placed to advocate for a holistic view of the overall assessment process. Nor are they likely to advocate for more meetings to discuss the difficulty of individual examination questions to ensure pass marks are set using a justified standard setting method.

How can the assessment theory-practice gap be bridged? Clearly, assessment practices vary across medical schools both nationally and internationally. In medical schools that perhaps do not have clearly established assessment practices ensuring continual review and refinement, or perhaps in those simply in need of an assessment refresh, we propose two key ways that can strengthen the theory-practice gap.

## Identify theory in the practice

What is the underlying theory in your school’s assessment strategy [7]? Perhaps it is underpinned by the psychometric model in which assessments of skills, knowledge and professionalism are taken as indicators of an individual student’s readiness to progress in each domain, and assessment quality is assured through measures of reliability and validity. Or perhaps your medical school has moved to a programmatic assessment strategy in which assessments serve as low stakes data points, which are then meaningfully combined to make a high stakes decision about the construct of medical student competence [8]. Either way, it is important to contemplate the overall purpose of each assessment, what the possible outcomes are (will there be a pass-fail decision?), and what they mean for the student when it comes to the high stakes decision. The answers to these questions will not only guide the standard setting strategy [2], but also the writing of assessment materials and ongoing evaluation of assessment items through metrics such as difficulty and discrimination indices [9]. A robust assessment system requires challenging assumptions at the program level (what are the underlying theoretical goals of our assessment system?), individual assessment level (what is the purpose of this assessment? what standard setting method will be used?), and individual assessment item level (what is the quality of each item? This might include metrics such as difficulty and discrimination indices or station level metrics in an OSCE [10]).

## Consider structural limitations to integrating theory and practice

The second suggestion is for medical schools to take a critical look at how their assessment teams are structured. Do they facilitate the blending of theory and practice? If researchers are rewarded only for research outputs, there is little incentive for them to be involved in the implementation of assessment practice. Meanwhile, those involved in operations are not likely rewarded for their involvement in or knowledge of current research. Integrating theory and practice might involve creating specialist positions specifically dedicated to quality assurance and improvement of assessment systems as a whole. Furthermore, busy physicians may not have the time to keep abreast of the latest assessment theories in addition to their clinical specialty (required for both teaching and practice). While perhaps unconventional, non-physician assessment experts might be well suited to lead assessment teams and oversee assessment practice. Finally, in some cases assessment may seem to be a purely administrative task that does not require much theoretical input. We believe this to be an oversimplification of a very important element of medical education. The importance of teamwork and inter-professional working is widely recognized throughout medical education, and this is true too of assessment systems and practices.

In conclusion, we argue that assessment research may be limited by a lack of integration between theory and practice. We have offered two suggestions for medical schools to consider; identifying and challenging the theory underlying current assessment practices, and considering structural limitations that may impede the integration of assessment theory and practice.
